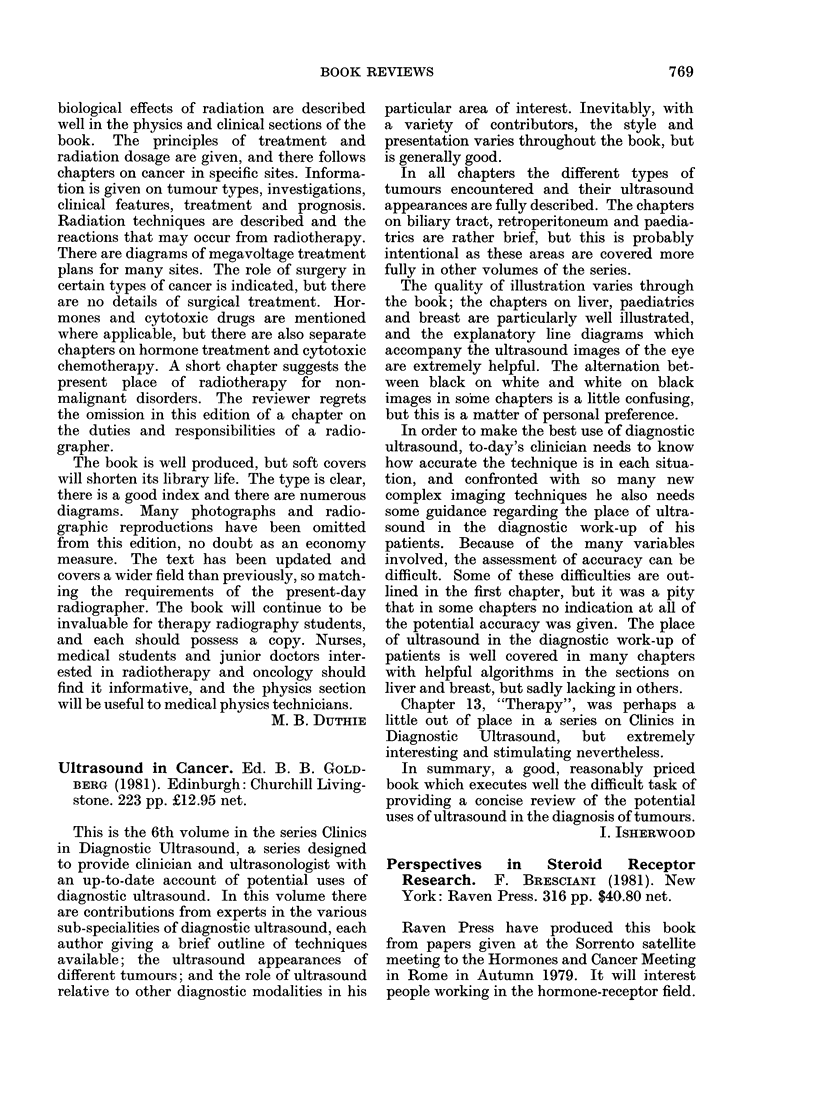# Ultrasound in Cancer

**Published:** 1981-11

**Authors:** I. Isherwood


**Ultrasound in Cancer.** Ed. B. B. Goldberg (1981). Edinburgh: Churchill Livingstone. 223 pp. £12.95 net.

This is the 6th volume in the series Clinics in Diagnostic Ultrasound, a series designed to provide clinician and ultrasonologist with an up-to-date account of potential uses of diagnostic ultrasound. In this volume there are contributions from experts in the various sub-specialities of diagnostic ultrasound, each author giving a brief outline of techniques available; the ultrasound appearances of different tumours; and the role of ultrasound relative to other diagnostic modalities in his particular area of interest. Inevitably, with a variety of contributors, the style and presentation varies throughout the book, but is generally good.

In all chapters the different types of tumours encountered and their ultrasound appearances are fully described. The chapters on biliary tract, retroperitoneum and paediatrics are rather brief, but this is probably intentional as these areas are covered more fully in other volumes of the series.

The quality of illustration varies through the book; the chapters on liver, paediatrics and breast are particularly well illustrated, and the explanatory line diagrams which accompany the ultrasound images of the eye are extremely helpful. The alternation between black on white and white on black images in some chapters is a little confusing, but this is a matter of personal preference.

In order to make the best use of diagnostic ultrasound, to-day's clinician needs to know how accurate the technique is in each situation, and confronted with so many new complex imaging techniques he also needs some guidance regarding the place of ultrasound in the diagnostic work-up of his patients. Because of the many variables involved, the assessment of accuracy can be difficult. Some of these difficulties are outlined in the first chapter, but it was a pity that in some chapters no indication at all of the potential accuracy was given. The place of ultrasound in the diagnostic work-up of patients is well covered in many chapters with helpful algorithms in the sections on liver and breast, but sadly lacking in others.

Chapter 13, "Therapy", was perhaps a little out of place in a series on Clinics in Diagnostic Ultrasound, but extremely interesting and stimulating nevertheless.

In summary, a good, reasonably priced book which executes well the difficult task of providing a concise review of the potential uses of ultrasound in the diagnosis of tumours.

I. Isherwood